# Comparative Study of Multiple High-Signal-Intensity Spots on 3D and 2D Magnetic Resonance Sialography for Patients with Sjögren's Syndrome

**DOI:** 10.1155/2021/5846637

**Published:** 2021-12-26

**Authors:** Yusuke Shimada, Ikuho Kojima, Masahiro Iikubo

**Affiliations:** Department of Dental Informatics and Radiology, Tohoku University Graduate School of Dentistry, 4-1 Seiryo-machi, Aoba-ku, Sendai 980-8575, Japan

## Abstract

We investigated the sensitivities of 2-dimensional (2D) magnetic resonance sialography (MR-S) and unilateral sagittal and axial 3-dimensional (3D) MR-S using a surface coil and their combination in diagnosing patients with Sjögren's syndrome (SS). We retrospectively analyzed the 3D and 2D MR-S results of 78 patients with SS. We evaluated the sensitivities of multiple high-signal-intensity spots and staging on MR sialograms and analyzed the efficient imaging methods and cross section for diagnosing patients with SS. The sensitivities of MR-S for detecting abnormal findings (i.e., MR-S stage 1 or higher) were as follows: 65 cases (83.3% [95% confidence interval (CI): 73.2–90.8]) for unilateral sagittal 3D MR-S; 62 cases (79.4% [95% CI: 68.8–87.8]) for axial 3D MR-S; 66 cases (84.6% [95% CI: 74.7–91.8]) for combined unilateral sagittal and axial 3D MR-S; and 32 cases (41.0% [95% CI: 30.0–52.7]) for bilateral sagittal 2D MR-S. The ratio of the abnormal finding of MR-S was tested using the two-tailed Fisher's exact test. Unilateral sagittal, axial, and combined unilateral sagittal and axial 3D MR-S showed significantly higher sensitivity than bilateral sagittal 2D MR-S, respectively (*P* < 0.001). Most cases upstaged by 3D MR-S were those positive (stage 1 or higher) among the stage 0 cases detected by 2D MR-S. Axial 3D MR-S, compared with 2D MR-S, understaged four cases, which was due to the imaging range of the axial 3D MR-S. We concluded that a single unilateral sagittal 3D MR-S was sufficient and axial 3D MR-S was unnecessary for SS staging. T1- and T2-weighted images are essential for investigating the salivary glands in patients with SS. Therefore, we also concluded that bilateral sagittal 3D MR-S of the parotid glands in addition to T1- and T2-weighted imaging is necessary, sufficient, and most efficient for precise MR imaging examination of the salivary glands, including diagnosing SS.

## 1. Introduction

Magnetic resonance (MR) imaging has become widely used for assessing salivary gland diseases and is useful for the diagnosis of Sjögren's syndrome (SS). X-ray sialography, whose apple tree appearance has been adopted for some SS criteria [1, 2], cannot simultaneously and bilaterally examine the three pairs of major salivary glands (i.e., parotid, submandibular, and sublingual glands) and is invasive and requires the use of contrast medium. Conversely, MR imaging, including MR sialography (MR-S), can bilaterally evaluate all major salivary glands simultaneously without contrast medium. MR imaging of patients with SS shows characteristic findings of the parotid gland, including a heterogeneous signal-intensity distribution (HD) on T1- and T2-weighted images (salt and pepper appearance) [3, 4], multiple high-signal-intensity spots (MHS) on MR-S (apple tree appearance) [5, 6], and swelling or atrophy of the parotid gland [4, 7]. Moreover, MR-S has a higher sensitivity (>82%) for the diagnosis of SS than conventional MRI, salivary gland scintigraphy, or ultrasonography [8–12]. Diffusion-weighted MRI also possessed only additional utility to MR-S in the diagnosis of SS [13]. Thus, MR-S is expected to be an alternative to conventional X-ray sialography for the diagnosis of SS.

Three-dimensional (3D) MR-S techniques facilitate the reliable prediction of ductal dilatation, stenosis, and damage better than the two-dimensional (2D) techniques [14–16]. Moreover, high-resolution MR-S using a small surface coil has been reported [17, 18]. MR-S using a combination of the constructive interference in steady-state sequence and the half-fourier acquisition single-shot turbo spin-echo sequence have been reported to be useful for patients with inflammatory salivary gland disorder [15]. However, these studies were mainly aimed at depicting detailed salivary gland ducts, and none of them mentioned the diagnostic accuracy of SS. Moreover, there is no study comparing the diagnostic accuracy of SS between 3D and 2D MR-S. The longer duration of acquisition (more than a few minutes per cross section imaging) and narrow range of acquisition (less than approximately 30 mm width) are major disadvantages of 3D MR-S for routine clinical use relative to 2D MR-S (imaging time less than approximately 20 s). Additionally, sagittal 3D MR-S of the bilateral parotid glands and axial 3D MR-S are time-consuming. The use of special coils, which are not used for other MR imaging techniques, such as high-resolution small surface coils, is also time-consuming and difficult. Furthermore, the contribution of 3D MR-S in combination with 2D MR-S to the sensitivity of the diagnosis of SS in individual patients has not been established. Therefore, we investigated the sensitivities of 2D MR-S and sagittal/axial 3D MR-S using a regular surface coil and their combination in diagnosing SS.

## 2. Materials and Methods

The procedures in this study adhered to the Helsinki Declaration of 1975, as revised in 1983. This retrospective study was approved by our institutional review board (no. 2012-24-1). Informed patient consent was obtained via an opt-out system on our institutional website displaying information about the study objectives and procedures.

### 2.1. Patients

We retrospectively reviewed the MR images of 78 patients (76 women and 2 men; age, 19–79 years; mean age, 55 years) clinically diagnosed with SS based on the chief complaint of xerostomia, positive histopathological findings of labial gland biopsy, and positive serologic evidence of anti-SS-A/Ro and/or anti-SS-B/La antibodies. Autoimmune disease specialists diagnosed all the patients with SS; the patients were negative for human immunodeficiency virus and sarcoidosis. We adopted labial gland biopsy as a criterion for all patients and graded the histopathological findings using focal scores according to previously established criteria [19]. We determined the histopathological findings based on the focal scores with focal aggregates of 50 lymphocytes per 4 mm^2^ of salivary gland tissue. Therefore, positive histopathological findings, the chief complaint of oral dryness, and anti-SS-A/Ro and/or anti-SS-B/La antibodies formed the basis of a definitive diagnosis of SS according to the criteria proposed by the American-European Consensus Group [1], American College of Rheumatology [20], and revised Japanese criteria [2].

### 2.2. MR Imaging Technique

We performed MR imaging with a 1.5- or 3.0-Tesla MR unit (Achieva 1.5 T or Achieva 3.0 T; Royal Philips Electronics, Eindhoven, the Netherlands) using the surface coils with a loop diameter of 11 cm. Transverse T1-weighted images (TR/TE, 477–619/7–12 ms) and fat-suppressed T2-weighted images (TR/TE, 4000–5711/54–90 ms) were obtained using the spin-echo and fast-spin-echo techniques, respectively. The section thickness of the transverse T1- and T2-weighted images was 5 mm with an intersection gap of 1 mm, acquisition matrix of 512 × 358, and field of view (FOV) of 210 mm. MR-S was performed using a high-resolution technique and a heavily T2-weighted 3D fast-spin-echo sequence (TR/TE/number of signal-intensity acquisitions, 5000/602–675 ms/2) according to a previous report that a heavily T2-weighted 3D fast-spin-echo sequence is the most suitable for the evaluation of thin salivary gland ducts (section thickness: 1 mm, gapless image, number of slices: 20, pixel size: 0.45–0.55 mm × 0.55–0.79 mm, FOV: 200 mm; scan time: 185 s) [21]. The 2D MR-S was performed using a single-shot 2D sequence (TR/TE, 8000–9000/800–914, the slice thickness: 30 mm, pixel size: 0.88 mm × 1.08 mm, FOV: 200 mm, scan time: 18–25 sec.). The MRI protocol for routine clinical practice was as follows: first, we performed T1- and T2-weighted imaging of the salivary glands, followed by the bilateral sagittal 2D MR-S of the parotid glands. Second, we performed axial 3D MR-S, which involved the bilateral parotid gland ducts. Finally, unilateral sagittal 3D MR-S was performed for the parotid gland. The laterality of the sagittal 3D MR-S was decided by the oral and maxillofacial radiologists based on the following: we selected the side that clearly showed the MHS on 2D MR-S. If MHS was not observed bilaterally, we assessed the T1- and T2-weighted images and proceeded with the side with strong HD findings.

### 2.3. Imaging Analysis and Sensitivity for Sjögren's Syndrome

**Figure 1:**
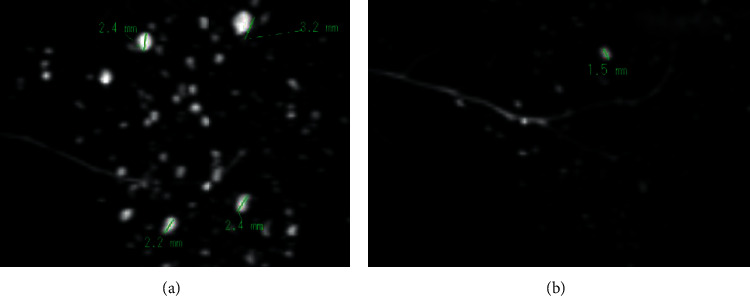
Analysis and measurement of MHS on MR-S. We staged the presence or absence of MHS and measured the size of the maximum diameter of the high-signal-intensity spots by the viewer software on display. A case of stage 3 with the maximum cavity >2 mm (a) and another case of stage 2 with the maximum cavity of 1-2 mm (b). MHS: multiple high-signal-intensity spots; MR-S: magnetic resonance sialography.

Two oral and maxillofacial radiologists with 11 and 19 years of experience, respectively, evaluated the coded and randomly presented unilateral sagittal 3D MR-S, axial 3D MR-S, and bilateral sagittal 2D MR-S for the presence or absence of MHS based on a previous report [6]. Both radiologists were blinded to the histopathological grading and T1- and T2-weighted imaging findings. The radiologists used digital imaging and communications in medicine viewer and processing software (nv-1000; Hitachi Medical Corporation, Tokyo, Japan) and were free to use the available image-enhancement options (density, contrast, and magnification) to perform visual assessments on the same 29.7-inch display (2560 × 1600 screen resolution, RadiForce RX440; EIZO Corporation, Ishikawa, Japan). Using the X-ray sialography criteria established in previous reports, we staged the MHS measured the size of the maximum diameter of the high-signal-intensity spots, which represented saliva leaked from peripheral ducts [10] by the viewer software on display (Figure 1). MHS was classified as stages 0 (no cavities found), 1 (cavities ≤1 mm in diameter), 2 (cavities 1-2 mm in diameter), 3 (cavities >2 mm in diameter), or 4 (severe irregular dilatation of the main duct with bizarrely patterned cavities). After an independent evaluation, the reviewers compared and discussed their staging. Any differences in interpretation were discussed, and a consensus was reached.

### 2.4. Statistical Analysis

Categorical variables are presented as counts and percentages. We determined the sensitivity of the MHS (according to previous reports [10], stage 0 is normal and stage 1 or higher is abnormal), and the sensitivity (ability to detect abnormalities) were compared between 3D MR-S and 2D MR-S for each sagittal image of 98 sides, and between the unilateral sagittal 3D MR-S, axial 3D MR-S, combined unilateral sagittal + axial 3D MR-S, and bilateral sagittal 2D MR-S patientwise in 78 patients, which were tested using the two-tailed Fisher's exact test. In case of significant association for categorical variables with a multiple comparison (patientwise comparison), pairwise comparison using adjustment methods of the Bonferroni correction was implemented. All *P* values were two-sided, and statistical significance was set at *P* < 0.05. Interobserver agreement between the two reviewers in the imaging analysis was evaluated using the kappa coefficient (ƙ). The strength of agreement was classified as slight (0.01 to 0.20), fair (0.21 to 0.40), moderate (0.41 to 0.60), substantial (0.61 to 0.80), or excellent (0.81 to 1.00). The sample size calculation for this study was based on Fisher's exact test for a power of 80% with reference to the accuracy of MR-S in a previous report [8]. A total of 92 samples would be required to test the association at 5% levels using a two-tailed test. EZR software (Saitama Medical Center, Jichi Medical University, Saitama, Japan), providing a graphical interface for *R* (v3.2.2; The *R* Foundation for Statistical Computing, Vienna, Austria), was used for statistical analyses.

## 3. Results

In the imaging analysis for the staging of MR-S, which indicates the detection of the MHS and measurement of the diameter of the MHS, the kappa values for the interobserver agreement of the sagittal 3D MR-S, axial 3D MR-S, and sagittal 2D MR-S were 0.92 (95% confidence interval [CI], 0.808–1.02), 0.96 (95% CI, 0.881–1.04), and 0.85 (95% CI, 0.744–0.957), respectively. All the values showed high agreement (excellent).

The MR-S staging on 98 sides of 78 patients with SS is shown in Table 1. The sagittal 3D MR-S identified MHS in 21 stage 0 cases, 40 stage 1 cases, 24 stage 2 cases, 12 stage 3 cases, and 1 stage 4 case. The sagittal 2D MR-S showed MHS for 58 stage 0 cases, 10 stage 1 cases, 22 stage 2 cases, 7 stage 3 cases, and 1 stage 4 case. Focusing on the frequencies of cases of stages 0–4, the 3D MR-S can detect more cases of stage 1 or higher than stage 0 than 2D MR-S. MR-S showed a statistically significant difference between the sensitivity for detecting abnormal findings of MHS between the two imaging methods: 78.6% (95% CI, 69.1–86.2%) on the 77 sides for 3D MR-S and 40.8% (95% CI, 31–51.2%) on the 40 sides for 2D MR-S (*P* = 0.000000106).

**Figure 2:**
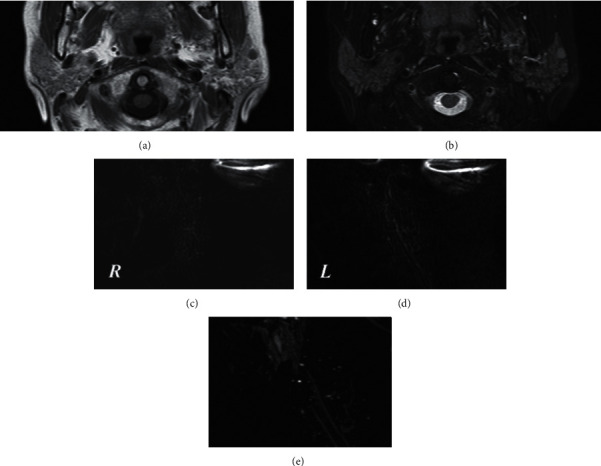
A case of the usefulness in determining the imaging side for unilateral sagittal 3D MR-S with T1- and T2-weighted images. T1-weighted image (a) and fat-suppressed T2-weighted image (b) showed HD of the parotid gland with left predominance. This case had no MHS in bilateral 2D MR-S (c, d). We determined the imaging side (right or left) for unilateral sagittal 3D MR-S by evaluating the HD on T1- and T2-weighted images (b, c). Unilateral left 3D MR-S (e) clearly depicted MHS. MR-S: magnetic resonance sialography; HD: heterogeneous distribution; MHS: multiple high-signal-intensity spots.

The patientwise MR-S staging for SS based on imaging findings and cross section is shown in Table 2. MR-S showed abnormal findings of MHS (i.e., stage 1 or higher): 65 cases (83.3%; 95% CI, 73.2–90.8%) for unilateral sagittal 3D MR-S; 62 cases (79.4%; 95% CI, 68.8–87.8%) for axial 3D MR-S; 66 cases (84.6%; 95% CI, 74.7–91.8%) for unilateral sagittal + axial 3D MR-S; and 32 cases (41.0%; 95% CI, 30–52.7%) for bilateral sagittal 2D MR-S. Unilateral sagittal, axial, and combined unilateral sagittal + axial 3D MR-S had significantly higher sensitivity than bilateral sagittal 2D MR-S (*P* = 0.00000043, *P* = 0.00000889, and *P* = 0.00000014, respectively). We checked the T1- and T2-weighted images and assessed the side with strong HD findings or tendencies if MHS was not observed bilaterally on bilateral 2D MR-S (Figure 2). As a result, the unilateral sagittal 3D MR-S images using this criterion had higher sensitivity than the axial 3D MR-S, including the bilateral parotid ducts.

**Figure 3:**
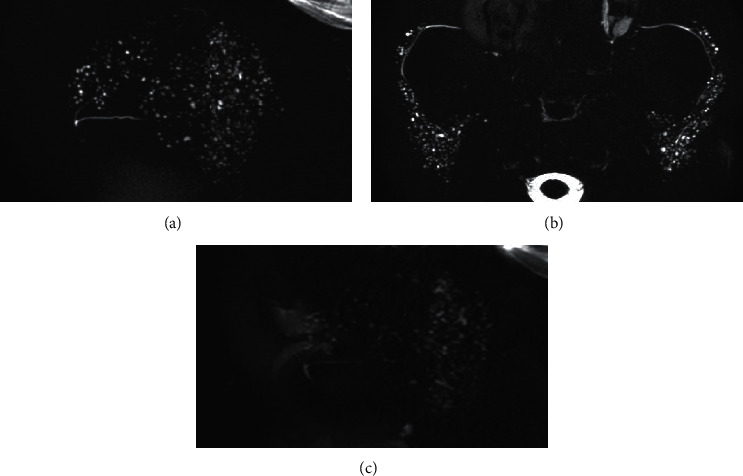
A case of detectable MHS on 3D and 2D MR-S. The sagittal 3D MR-S (a), axial 3D MR-S (b), and sagittal 2D MR-S (c) detected MHS and graded it as stage 2. MHS: multiple high-signal-intensity spots; MR-S: magnetic resonance sialography.

**Figure 4:**
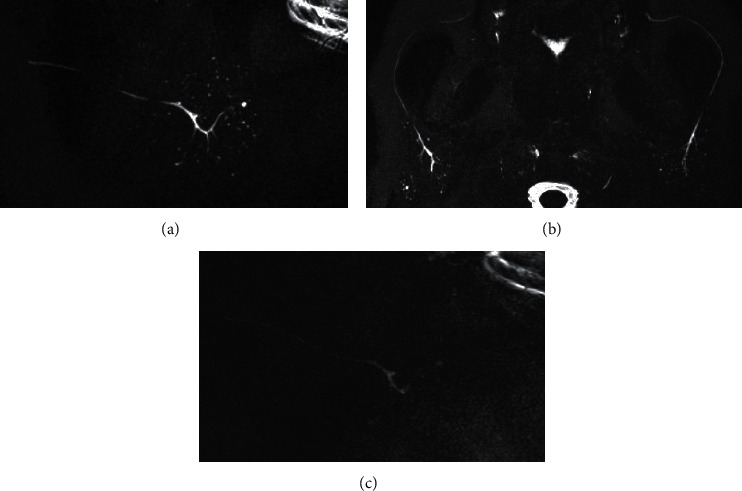
A case of detectable MHS only by 3D MR-S. The sagittal 3D MR-S (a) and axial 3D MR-S (b) were able to detect MHS and graded it as stage 1. The sagittal 2D MR-S (c) was not able to detect MHS and graded it as stage 0. MHS: multiple high-signal-intensity spots; MR-S: magnetic resonance sialography.

**Figure 5:**
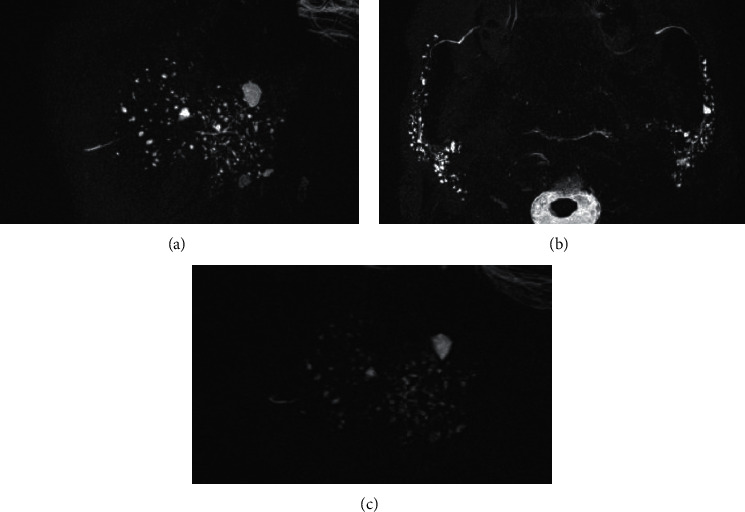
A case of understaging by axial 3D MR-S compared with the staging by 2D MR-S. Sagittal 3D (a), axial 3D (b), and sagittal 2D MR-S can all detect MHS. While the axial 3D MR-S (b) result was stage 2, sagittal 3D (a) and 2D (c) MR-S results indicated stage 3. The axial 3D MR-S (b) was not able to detect a larger size of MHS due to being out of imaging range. MR-S: magnetic resonance sialography; MHS: multiple high-signal-intensity spots.

Most cases designated as stage 2 or higher based on 3D MR-S could also be designated as stage 1 or higher by 2D MR-S (Figure 3). Most cases upstaged by 3D MR-S could be detected as positive (stage 1 or higher) from stage 0 by 2D MR-S (Figure 4). The unilateral sagittal, axial, and combined unilateral sagittal + axial 3D MR-S detected (upstaging) 33, 30, and 33 positive cases of stage 1 or higher, respectively, from stage 0 cases of 2D MR-S. The unilateral sagittal and axial 3D MR-S understaged 1 and 4 cases, respectively, compared to the staging of 2D MR-S. Some cases of understaging by axial 3D MR-S were due to the imaging range of MR-S (Figure 5). The one case of understaging by unilateral sagittal 3D MR-S was due to MHS on the opposite side.

## 4. Discussion

A comparison of the 3D and 2D techniques for sagittal MR-S (78 patients, 98 sides: Table 1) showed that 3D sagittal MR-S had significantly higher sensitivity than 2D MR-S. Similarly, unilateral sagittal, axial, and combined sagittal + axial 3D MR-S had significantly higher sensitivity for detecting SS-related findings than bilateral 2D MR-S by patientwise MR-S staging (Table 2). Three-dimensional MR-S enables the precise assessment of salivary gland ductal systems and detects early stages of SS with 91–100% accuracy [5]. Takagi et al. [18] demonstrated high diagnostic performance with MRI using a 47 mm microscopic coil, with an area under the curve of 0.91 in the receiver operating characteristics curve analysis for MR-S lesion counts. The ability of 3D MR-S to detect ductal structures is better than that of 2D MR-S, and the sensitivity is much better than the results of this study. The present findings indicated that 3D MR-S without the high-resolution coil and using a normal surface coil had a relatively high sensitivity for SS diagnosis, which was significantly superior to 2D MR-S. Although a simple comparative study between 2D and 3D methods would not be useful, this study examined the combination of 2D and 3D MR-S for efficient imaging by considering the imaging duration and cross section and the number of images.

Our results indicate that unilateral sagittal 3D MR-S is comparable to or better than axial 3D MR-S in diagnosing SS (Table 2). The axial 3D MR-S was unable to detect MHS at the upper and lower ends of the parotid gland for several cases because the imaging range was limited to 30 mm. In one case, unilateral sagittal 3D MR-S was unable to detect MHS because of presence on the opposite side. Moreover, bilateral sagittal MRI is not only recommended for MR-S-based staging but also for the diagnosis of the bilateral parotid duct because it covers both sides of the parotid gland within the imaging range. Kojima et al. [8] analyzed the diagnostic performance of MR imaging and MR-S of the three major salivary glands for SS and demonstrated low sensitivity and high specificity for the submandibular glands and low diagnostic performance for the sublingual gland. For detecting SS, MR-S of other salivary glands is not necessary. 3D MR-S of the parotid gland is sufficient. Several cases undergoing MR-S with 3D and high-resolution imaging using special coils have been reported [14–18]. However, no study has examined the combination of imaging methods that are efficient in terms of imaging duration, imaging cross section, and the number of images for each case. We concluded that a single unilateral sagittal 3D MR-S was sufficient and axial 3D MR-S was not necessary for SS staging.

In the abovementioned study that examined the diagnostic accuracy of SS [8], the MHS findings on MR-S and HD findings on T1- and T2-weighted images were compared. The results demonstrated that the MHS of MR-S of the parotid gland was the most sensitive at 82%, followed by the HD findings on T1- and T2-weighted images of the parotid gland, and then the HD findings of the submandibular gland, at 67% and 58%, respectively. Furthermore, the HD findings on T1- and T2-weighted images have been reported to be more greatly associated with the salivary secretion in patients with SS than MHS findings on MR-S [9]. In the present study, we checked the T1- and T2-weighted images and assessed the side with strong HD findings or tendencies if MHS was not observed bilaterally on bilateral 2D MR-S. The unilateral sagittal 3D MR-S images using this criterion had higher sensitivity than the axial 3D MR-S, including the bilateral parotid ducts. Therefore, considering the usefulness of the diagnostic value by previous reports and HD findings by the present study, T1- and T2-weighted images are essential for investigating the salivary glands in patients with SS.

Several stages of SS by 3D MR-S (unilateral sagittal, axial, and unilateral sagittal + axial) were upstaged from 2D MR-S. Most of them converted to stage 1 or more from stage 0 designated by 2D MR-S: unilateral sagittal 3D MR-S, 33 cases; axial 3D MR-S, 30 cases; and unilateral sagittal + axial 3D MR-S, 33 cases, respectively. In other words, because 2D MR-S often provides false-negative results, additional 3D MR-S imaging is necessary. A few 3D MR-S cases were understaged from the stage for SS on 2D MR-S: 3 cases by axial 3D MR-S and one case by unilateral sagittal 3D MR-S. The axial 3D MR-S results for some of these cases were due to the imaging range of MR-S, as described above. In the one unilateral sagittal 3D MR-S case, MHS could not be detected on the opposite side, which was due to the imaging range of MR-S. In addition, in the longitudinal parotid gland, the MHS can be easily distinguished from the thin parotid duct on sagittal 3D MR-S. Conversely, in axial 3D MR-S, the MHS and relatively thick parotid ducts are densely packed and overlap, making it difficult to distinguish between them, which is considered to result in understaging. Some cases of understaging by unilateral sagittal and axial MR-S may be misled as upstaging on 2D MR-S because of the partial volume effect on 2D MR-S with poor spatial resolution.

A limitation of this study was that we could not compare the sensitivities for detecting abnormal findings of the MHS for unilateral and bilateral 3D MR-S. Since we did not perform bilateral sagittal 3D MR-S and axial 3D MR-S at our hospital because of the limited imaging duration, the imaging side for sagittal 3D MR-S was decided during the examination by the oral and maxillofacial radiologists.

Another limitation of this study is the further improvement of the MR-S imaging technique, which should be considered in future studies. The development of imaging methods using phantoms of a wide range of imaging thickness and shorter duration of the imaging acquisition while maintaining the MR-S image quality and evaluating the diagnostic accuracy of these improved MR-S techniques is warranted.

## 5. Conclusions

We concluded that a single unilateral sagittal 3D MR-S was sufficient and axial 3D MR-S was not necessary for SS staging. T1- and T2-weighted images are essential for investigating the salivary glands in patients with SS. Therefore, we also concluded that bilateral sagittal 3D MR-S of the parotid glands in addition to T1- and T2-weighted imaging is necessary, sufficient, and most efficient for precise MRI examination of diagnosing SS.

## Figures and Tables

**Table 1:** MR-S staging by sagittal images of 98 sides.

	Stage 0	Stage 1	Stage 2	Stage 3	Stage 4	Sensitivity (%) [95% CI]	
3D MR-S (sagittal)	21	40	24	12	1	78.6 [69.1–86.2]	^*∗∗∗*^ *P* < 0.001^a^
2D MR-S (sagittal)	58	10	22	7	1	40.8 [31.0–51.2]	

^*∗∗∗*^, *P* < 0.001, comparison of sensitivities of 3D and 2D MR-S by ^a^Fisher's exact test. MR-S: magnetic resonance sialography; CI: confidence interval.

**Table 2:** Patientwise MR-S staging stratified by imaging method and cross section.

	Stage 0	Stage 1	Stage 2	Stage 3	Stage 4	Sensitivity (%) [95% CI]	
3D MR-S (unilateral sagittal)	13	35	20	9	1	83.3 [73.2–90.8]	^*∗∗∗*^ *P* < 0.001^a^ (vs. 2D MR-S)
3D MR-S (axial)	16	31	24	6	1	79.4 [68.8–87.8]	^*∗∗∗*^ *P* < 0.001^a^ (vs. 2D MR-S)
3D MR-S (unilateral sagittal + axial)	12	33	22	10	1	84.6 [74.7–91.8]	^*∗∗∗*^ *P* < 0.001^a^ (vs. 2D MR-S)
2D MR-S (bilateral sagittal)	46	6	20	5	1	41.0 [30.0–52.7]	

^*∗∗∗*^, *P* < 0.001, multiple comparisons of sensitivities by ^a^Fisher's exact test and pairwise comparisons with the Bonferroni correction. MR-S: magnetic resonance sialography; CI: confidence interval.

## Data Availability

Data can be provided upon request to the corresponding author.
